# Histology of orchidectomy specimens in Nigerian patients with prostrate cancer

**DOI:** 10.1186/1750-9378-6-S1-A3

**Published:** 2011-08-11

**Authors:** E Oluwabunmi Olapade-Olaopa, Ademola A Popoola, Alvan K Ukachukwu, Abimbola AA Oyelekan, Adeniyi Amos, Olayiwola B Shittu, Linus I Okeke

**Affiliations:** 1 Departments of Surgery, College of Medicine, University of Ibadan, Nigeria; 2 University College Hospital, Ibadan, Nigeria; 3 University of Ilorin Teaching Hospital, Ilorin, Nigeria

## Background

Carcinoma of the prostate (CaP) is the most common malignancy in men and its highest incidence, morbidity and mortality has been recorded in blacks. Bilateral orchidectomy remains the most common treatment for CaP in Sub-Saharan Africa. However, most of these patients respond poorly to this treatment especially in the presence of neurological deficits. It has been reported that CaP patients with testicular atrophy respond poorly to therapeutic orchidectomy. Testicular atrophy may therefore contribute to the observed poor response in our CaP patients to orchidectomy.

## Objective

To determine the prevalence of testicular atrophy in patients undergoing bilateral orchidectomy as primary treatment for CaP in two major teaching hospitals in Nigeria, as a first step to evaluating its prognostic value in men of this sub-population.

## Materials and methods

One hundred and forty-two (142) patients with histologically diagnosed prostate cancer had bilateral orchidectomy as their primary management modality between January 2001 and December 2008. The orchidectomy specimens were studied by experienced pathologists and the reports were analysed retrospectively. The criteria evaluated were: age, testicular weight, histological diagnosis, and the degree of atrophy per predetermined histological criteria. The testes were then classified as being normal, mildly, moderately or severely atrophic.

## Results

The mean age of the patients was 68.2 years and median age 69 years (range 27 – 92 years). Two (2) specimens (1.4%) had evidence of testicular metastases while 106 (74.6%) showed evidence of testicular atrophy and 34 (23.9%) of the specimens were reported as normal testes. Of the 106 specimens reported as atrophic, 33 (23.2%) showed mild atrophic changes and 73 (51.4%) showed moderate to severe atrophic changes. Six of the 142 orchidectomy specimens (4.2%) had different histology reports for the two testes.

## Conclusion

Majority of bilateral orchidectomy specimens from our CaP patients show variable degrees of atrophy whilst testicular metastases are rare. This finding may explain, in part, the poor response to surgical hormonal ablation observed in CaP patients in our community and is in keeping with the ealier studies. As such, urologists would have to develop protocols that provide for early recognition and care of post-orchidectomy hormone refractory disease.

## Acknowledgements

Publication of this article was funded in part by the University of Florida Open-Access Publishing Fund.

